# Simple Rectangular Gratings as a Near-Field “Anti-Reflection” Pattern for GaSb TPV Cells

**DOI:** 10.1038/s41598-017-01197-2

**Published:** 2017-04-21

**Authors:** Haitong Yu, Dong Liu, Zhen Yang, Yuanyuan Duan

**Affiliations:** 1 Key Laboratory of Thermal Science and Power Engineering of Ministry of Education, Beijing Key Laboratory for CO2 Utilization and Reduction Technology, Tsinghua University, Beijing, 100084 China; 2 MIIT Key Laboratory of Thermal Control of Electronic Equipment, School of Energy and Power Engineering, Nanjing University of Science and Technology, Nanjing, 210094 China

## Abstract

We show theoretically that 2D rectangular gratings on the surface of GaSb can serve as an “anti-reflection” pattern for nano-gap thermophotovoltaic (TPV) devices, which significantly enhances near-field radiative flux from the emitter to a GaSb cell, thus improving output power and conversion efficiency. The system in this study is a 200-nm gap TPV power generation system with a planar infrared plasmonic emitter and GaSb cell. Rigorous coupled-wave analysis is used to calculate the spectral near-field radiative flux involving periodic structures. The simulation shows that when coupled with a near-infrared plasmonic bulk emitter, adding gratings on the GaSb cell surface results in strong spectral enhancement above the cell’s bandgap and suppression for low-energy photon transmission, an effect that cannot be fully predicted by the effective medium theory. The resultant peak spectral heat flux is 2.8 times as high as the case without surface structures and the radiative transfer efficiency increased to 24.8% from the original 14.5% with the emitter temperature at 1800 K. The influence of the grating’s geometry parameters on the enhancement and peak frequency is further discussed with rigorous calculation of the spatial distribution of thermal radiative transfer that provided insight into the physical mechanism.

## Introduction

A TPV device generates electricity by converting the photons emitted from the high-temperature emitter through a low-bandgap PV cell, usually composed by group III–V semiconductor materials. The emitter can be heated by a wide range of energy sources including industrial waste heat^1^, solar energy^2^, and micro radioisotopes^3^. Therefore, TPV systems promise quiet, high-efficiency power conversion for a wide range of applications^4^. A nano-gap TPV, where the emitter and the cell are separated by a sub-micrometer gap, has the potential for significantly boosting the output power by making use of the evanescent component of the thermal radiative transfer, known as the near-field radiative transfer^5, 6^. Experiments have shown that the short circuit current of a TPV device increased dramatically when the gap was reduced to 1.8 μm^7^.

A critical issue in TPV devices is the spectral control of thermal radiation. The thermally emitted photons with energy lower than the cell’s bandgap cannot be converted to electrons, and photons with much higher frequency than the bandgap cannot be converted with full efficiency. Both cases will result in a loss of efficiency and hazardous heating of the cell. Therefore, the aim is to selectively achieve high spectral radiative heat flux slightly above the cell’s bandgap. Although the manipulation of near-field spectral radiative flux has been demonstrated in many systems, practical designs aimed at TPV systems are still scarce, mainly because group III–V semiconductors, typically GaSb, used as TPV cells have large refractive indices in the near to mid-IR spectral region and lack any plasmonic properties, which results in high Fresnel reflection coefficients and makes them poor thermal absorbers^8^. As the near-field spectral heat flux is determined by the coupling optical properties of materials on both sides, the weak absorption of GaSb greatly adds to the difficulty of spectral design to improve the output power and efficiency of a near-field TPV system. Although other types of materials with PV potential and higher absorption have been proposed as more ideal TPV cell candidates^8^, such as carbon nanotube, this strategy is not currently practical, and present TPV designs are still mostly aimed at group III–V semiconductors. Chang *et al*.^9^ paired a nanowire hyperbolic metametarial (HMM) emitter and a thin-film InGaSb cell separated by a 20-nm vacuum gap. Calculation using the effective medium theory (EMT) implicated enhanced radiative flux above the cell’s bandgap due to the HMM emitter and suppressed long wavelength radiative transfer due to the cell’s total internal reflection. Tong *et al*.^10^ suggested using ultrathin semiconductor films with perfect reflectors as thermal emitters and PV cells that act as a “thermal well” by confining photons in trapped waveguide modes to create selectively high spectral radiative flux between the emitter and the cell. Karalis *et al*.^11^ proposed a “squeezing” method to harvest near-field radiative energy by coupling an infrared plasmonic emitter and an ultrathin film cell, where the spectral range of radiative flux is confined by choosing the plasma frequency of the emitter and the waveguide modes inside the thin-film cell. The studies listed above focused on replacing the bulk semiconductor with thin-film cells to limit low frequency radiative heat flux as well as design the emitter materials accordingly to achieve high radiative flux above the cell bandgap, which might include the attempt to apply microstructures on the emitter side. Yet there has been little research on the effect of applying microstructures to the semiconductor cell.

For solar PV conversion and far-field TPV systems, various micro-structures have been designed for the semiconductor cell as surface anti-reflection patterns to improve the absorption. However, similar designs have yet to be studied in a nano-gap TPV system, not only because of the difficulty in calculating the near-field radiative flux involving structured objects, but also due to the increasing complexity of physical mechanisms. As a result, far-field anti-reflection strategies may fail to apply to near-field radiative transfer and vice versa. For example, Ijiro *et al*.^12^ showed experimentally that far-field anti-reflection micro-cavities suppressed the near-field radiative transfer as the gap decreased to sub-micron sizes. Chalabi *et al*.^13–15^ presented simulations showing that 1D gratings on SiC increased the near-field radiative flux and reduced the far-field radiative transfer. These very different radiative transfer behavior characteristics between the near-field and far-field effects are mainly due to the participation of evanescent waves with large lateral wave vectors, which do not exist in far-field scenarios, and due to the path of the radiative transfer becoming comparable to the characteristic size of the microstructures, which means that the addition of surface structures may significantly change the effective gap distances between two objects and also alter the spectral and spatial patterns of the radiative transfer for nano-size gaps.

Therefore, the aim of this work is to develop a near-field “anti-reflection” surface structure, which is analogous to its far-field counterparts in suppressing the semiconductor absorber’s reflection and enhancing the heat flux above the cell’s bandgap and remains effective for near-field setups by considering a sub-micron radiative transfer path and the participation of evanescent waves. We choose one of the most simple surface structures, a 2D rectangular grating, which has been shown to improve the absorption of bulk GaSb in the far-field regime^16^. Recent studies have further demonstrated the capability of gratings on significantly changing the spectral energy distribution of near-field radiative transfer between metals^17–19^, doped-Si^20^, and polar materials^21, 22^. However, adding gratings to a group III–V semiconductor as absorbers has not been discussed thus far. For the thermal emitter, an infrared plasmonic material (GZO) is used due to its ability to excite strong surface waves matching the cell’s bandgap and thus enhancing near-field radiative transfer by frustrated modes. The studied nano-gap TPV system has a vacuum gap of 200 nm, which is currently experimentally practical^23, 24^. Rigorous coupled-wave analysis (RCWA), a commonly used method to analyze grating optical properties, is extended into the regime of near-field radiative transfer via fluctuational dynamics and scattering matrices. The results with gratings added are compared to planar setups and the physical mechanisms are explained.

## Results

### Material choice and grating structure

**Figure 1:**
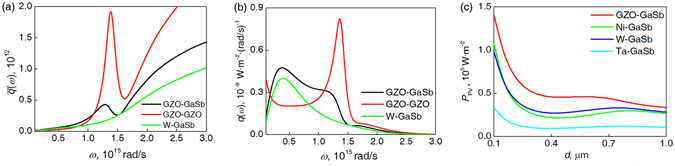
Analytical result of the radiative transfer between GZO and GaSb in comparison to other setups. (**a**) Normalized spectral radiative flux for vacuum gap *d* = 200 nm. (**b**) Spectral radiative flux from an 1800 K emitter to a 300 K GaSb at *d* = 200 nm. (**c**) The manifested electrical power *P*_PV_ from an 1800 K emitter to a 300 K GaSb cell at various vacuum gaps.

The calculated system of near-field radiative transfer consists of 2 parts, the thermal emitter and the absorber. The absorber is a bulk GaSb cell with a bandgap of 0.726 eV (*ω*_g_ = 1.1 × 10^15^ rad/s). The optical parameters of GaSb are calculated using a universal model for III–V semiconductors^25^. The emitter was chosen to be a plasmonic material with its plasmonic frequency slightly above the cell’s bandgap, which enables strong surface wave excitations in the designated frequency range to maximize the radiative transfer efficiency^11^. In this work, the optical parameters are taken from the experimental data of Ga:ZnO (GZO)^26^, which has an near-infrared (NIR) plasmonic frequency (1.38 × 10^15^ rad/s) and high melting point to suit the requirement for a TPV emitter. Figure 1 shows the analytical result of radiative heat flux between bulk GZO and GaSb in comparison to other setups, calculated by Equations (1)–(6) in Methods. Compared to refractory metallic emitters, using an NIR plasmonic emitter enables selectively higher spectral radiative flux above the cell’s bandgap, which results in higher effective radiative flux (defined in Methods) at sub-micron vacuum gaps. However, the spectral selectivity of the GZO-GaSb pair is still significantly inferior when compared to the GZO-GZO pair due to GaSb’s high reflection coefficients with no matching plasmonic properties with the emitter.

**Figure 2:**
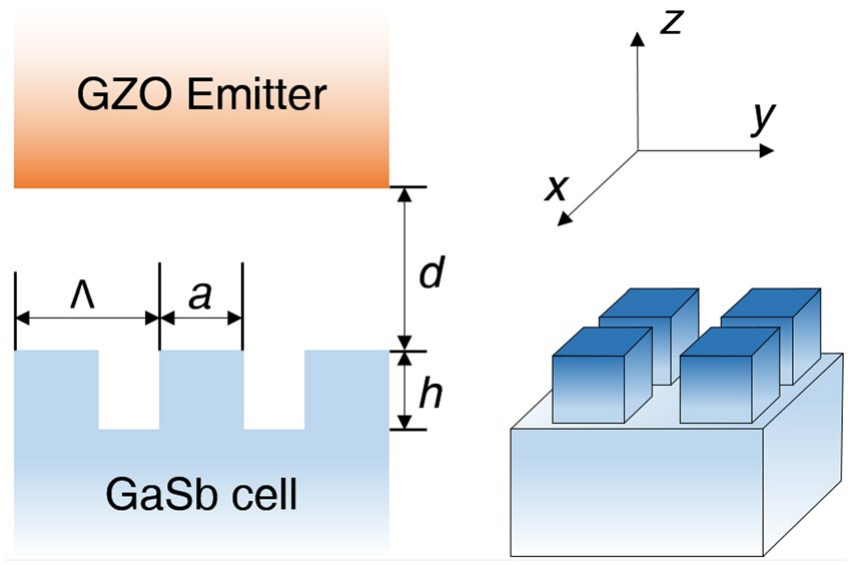
Illustration of the geometry configuration, the structural parameters, and the assigned coordinates.

To suppress the GaSb absorber’s reflection, 2D rectangular grating structures are added to the top surface of GaSb as a near-field “anti-reflection” structure (Fig. 2), which is determined by 3 geometry parameters, the period Λ, the ridge length *a*, and the channel depth *h*. Character sizes along the *x* and *y* direction are the same. The filling ratio *f* of the grating is *f* = (*a*/Λ)^2^. The separation distance between the top surfaces of GZO and GaSb is *d* = 200 nm.

### Spectral enhancement of radiative flux

**Figure 3:**
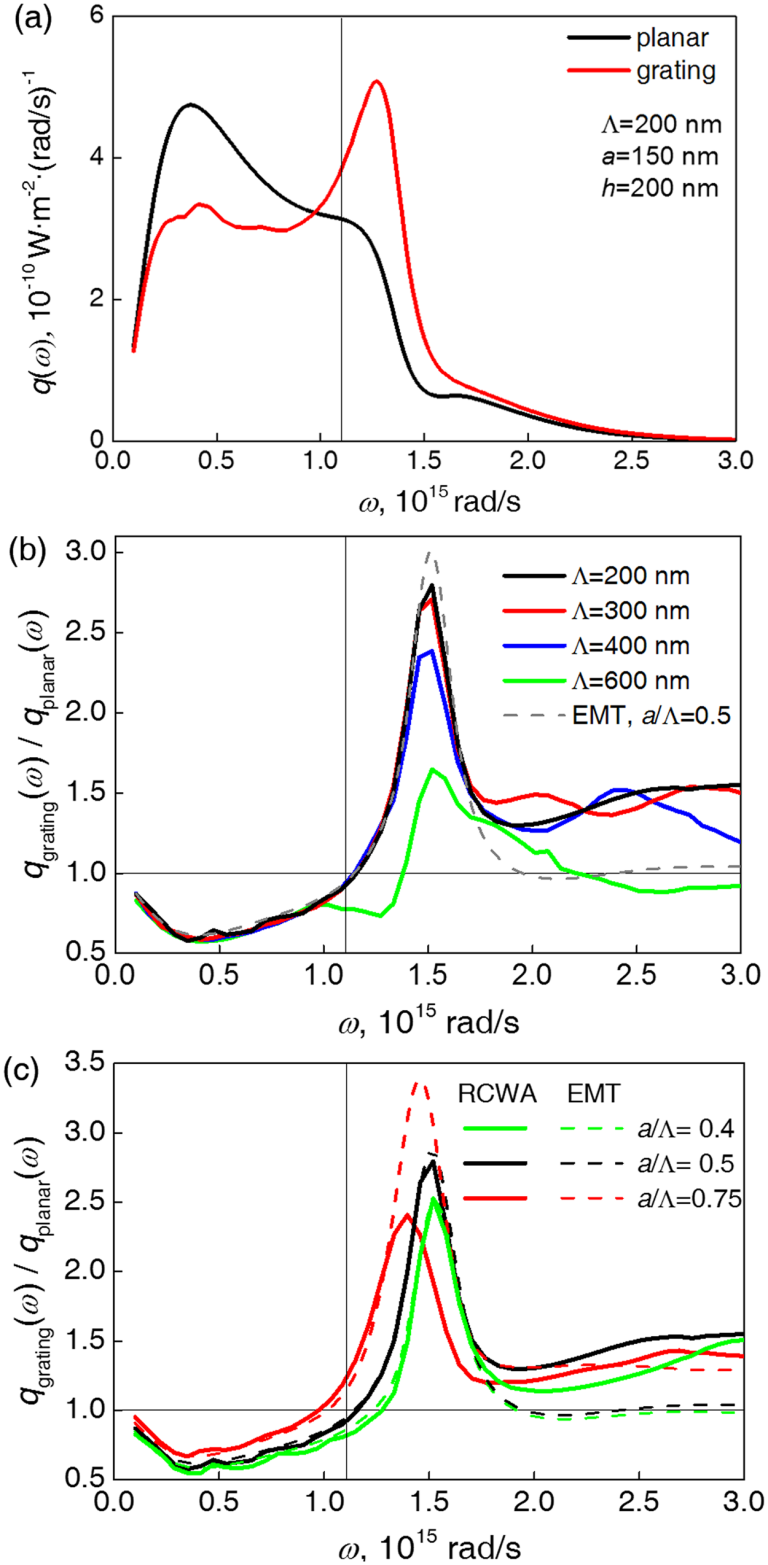
(**a**) Comparison of spectral radiative flux *q*(*ω*) for GaSb absorber with and without surface gratings (structural parameters shown in figure). (**b**) Influence of grating period, Λ, with fixed *a*/Λ = 0.5 and *h* = 200 nm. (**c**) Influence of grating filling ratio, with fixed Λ = 200 nm and *h* = 200 nm. The corresponding EMT results are shown in color-matched dashed lines and the GaSb bandgap *ω*_g_ is marked.

The simulation deploys the RCWA method integrated with fluctuational dynamics and scattering matrix method (see the description and validation in Methods). The impact of adding surface grating structures to GaSb on radiative flux *q*(*ω*) is shown in Fig. 3(a). Compared to the planar case, GaSb with rectangular grating structures shows significantly higher absorption of radiative energy above *ω*_g_ and suppressed radiative transfer below it, thus proving the selective anti-reflection effect of the nano-patterning.

To show the influence of structural parameters with better clarity, Fig. 3(b) and (c) show the ratio of spectral radiative flux in the grating case, *q*_grating_(*ω*) and the planar case, *q*_planar_(*ω*). Considering the small characteristic length of the gratings used, EMT calculations^27, 28^ are also plotted as reference results. Figure 3(b) shows the influence of Λ when the filling ratio and grating height is fixed. At *a*/Λ = 0.5, the frequency for maximum enhancement is 1.5 × 10^15^ rad/s and remains unchanged with varying Λ. As the period decreases from 600 nm, the peak spectral enhancement keeps increasing until Λ reaches 200 nm, when the spectral radiative flux also approaches the prediction by EMT, with a maximal relative enhancement of 2.8 times the planar setup. Reducing the period further shows no significant change in spectral patterns. The results show the peak frequency is mainly determined by *a*/Λ, which can also be approximately predicted by EMT, although for longer periods, the influence of inhomogeneity becomes stronger, which breaks the continuity for lateral wave propagation in the GaSb surface layer and causes the different result from EMT.

Figure 3(c) shows the influence of *a*/Λ on the peak frequency with a fixed period. As *a*/Λ increases, the peak enhancement shifts toward a lower frequency, which agrees with the prediction by EMT; yet, for larger filling ratios where the aspect ratio of the grating channels becomes higher, the rigorous results deviated from EMT’s estimation, even when the characteristic size in question remained small enough compared to the studied wavelength. For longer period, this deviation became more apparent, as shown in Fig. S3 in Supplementary Materials with Λ = 400 nm.

In all studied cases, the radiative flux is generally enhanced above GaSb’s bandgap and somewhat suppressed below it, which altogether shows that the manipulated spectral radiative flux is in favor of higher TPV conversion efficiency.

### Distribution of radiative energy along wave vector

**Figure 4:**
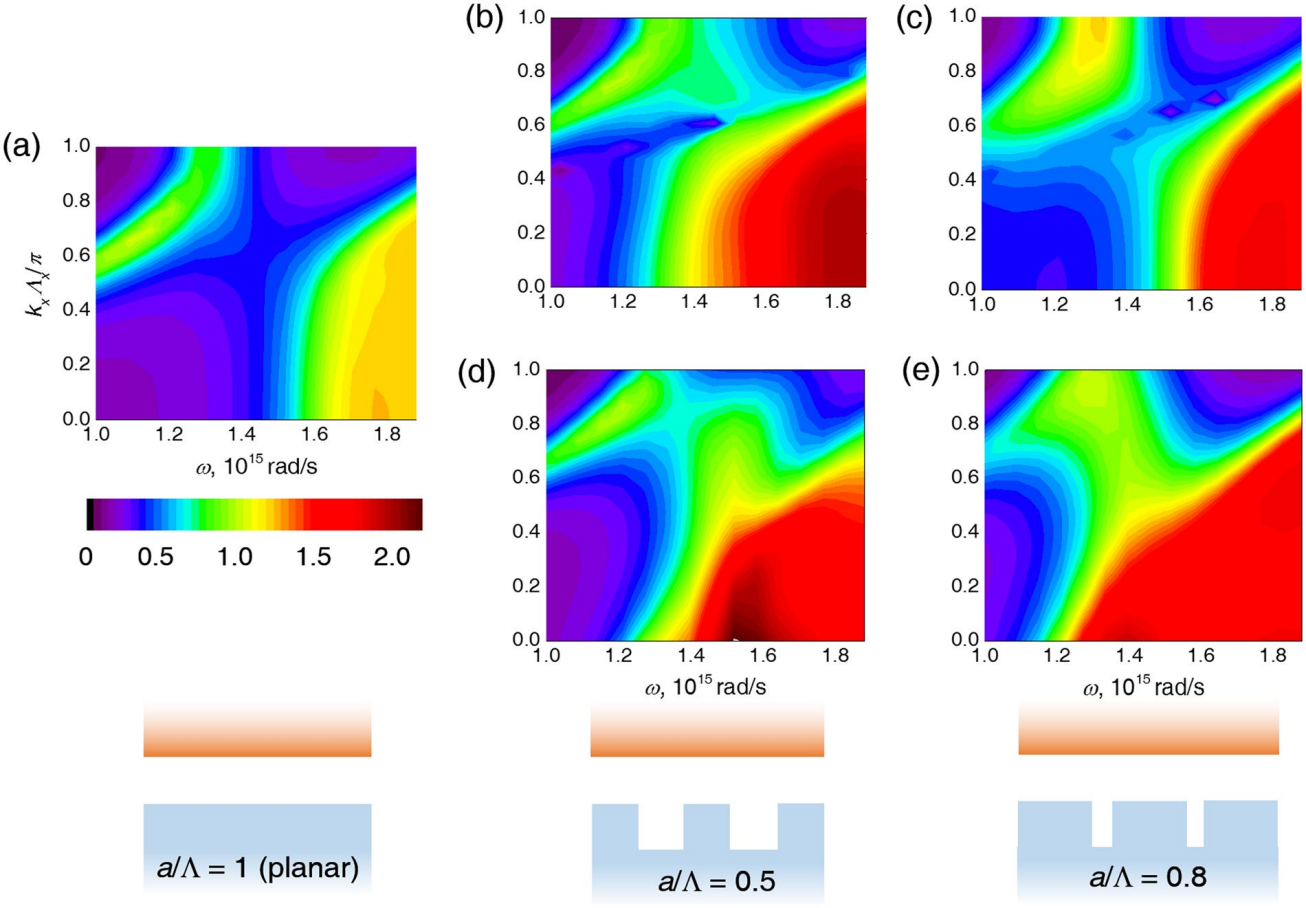
*k*_*x*_-*ω* distribution of energy transmission coefficient *ξ*(**k**, *ω*) (for *k*_*y*_ = 0) for Λ = 400 nm and *h* = 200 nm. (**a**) Planar. (**b**) *a*/Λ = 0.5. (**c**) *a*/Λ = 0.8. (**d**) and (**e**) show the EMT result corresponding to (**b**) and (**c**).

Figure 3 shows that for a short period length and low *a*/Λ, rigorous calculation yields very similar results to EMT, suggesting that the surface grating can well be characterized by a homogenous layer with effective indices, which reduces the reflection coefficient by significantly lowering the refractive indices (Figure S2). To explain the enhancement by gratings with larger *a*/Λ and also the deviation from EMT, we studied the distribution of radiative energy along plane wave vector **k** by showing the transmission coefficient *ξ*(**k**, *ω*) calculated by a rigorous method and EMT in Fig. 4. Note that because RCWA only deals with the first Brillouin zone and uses wave expansion to account for the wave vectors out of this region, the resulting *ξ*(**k**, *ω*) is the sum of the transmission coefficient for all **k** = **k**_**0 **_ ± (2*mπ*/Λ, 2*nπ*/Λ) where *m* and *n* are integers and can be larger than 1. The results for planar cases and by EMT are also processed accordingly for better clarification.

Compared to the planar setup shown in Fig. 4a, gratings with various filling ratios generally result in enhanced *ξ*(**k**, *ω*) for frequencies above GaSb’s bandgap, but in different sections in the *k*-*ω* plot, determined by the grating filling ratio. For *a*/Λ = 0.5, RCWA and EMT derives a similar distribution of radiative energy, where the major enhancement is located at smaller *k*_*x*_ toward the higher frequency, suggesting that the gratings with lower aspect ratios share similarity with a homogeneous layer of effective medium. Gratings with higher filling ratios have different results, as Fig. 4(c) shows a major enhancement of transmission factor at *k*_*x*_Λ_*x*_/*π* → 1 (grating channels) at 1.3 × 10^15^ rad/s, which is not predicted by EMT. This suggests that in this case, the grating channels create stronger resonance than the lower filling ratio case, which resulted in enhanced radiative flux and cannot be fully predicted by the EMT calculation.

**Figure 5:**
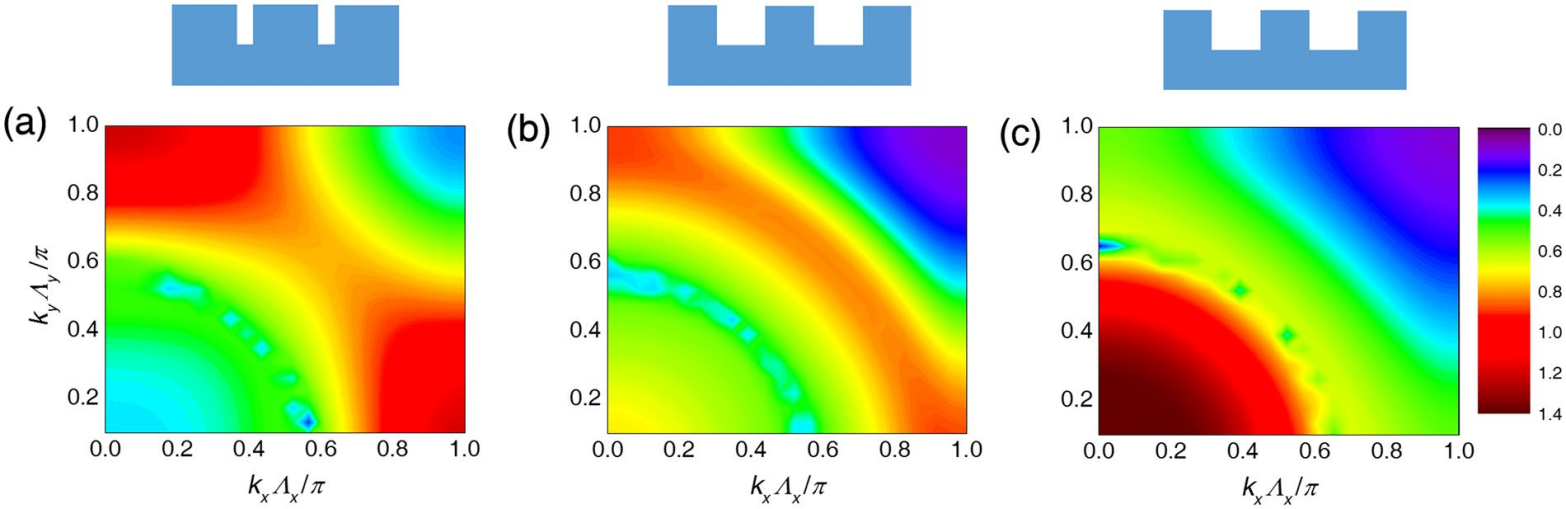
*k*_*x*_-*k*_*y*_ distribution of the energy transmission coefficient *ξ*(**k**, *ω*) at peak frequency for Λ = 400 nm and *h* = 200 nm. (**a**) *a*/Λ = 0.8 at 1.3 × 10^15^ rad/s. (**b**) *a*/Λ = 0.5 at 1.3 × 10^15^ rad/s. (**c**) *a*/Λ = 0.5 at 1.5 × 10^15^ rad/s.

Figure 5 further explains the different physical mechanisms for grating enhancement by plotting *ξ* along *k*_*x*_ and *k*_*y*_ for a single frequency. Corresponding to Fig. 4, the results show that gratings with higher size aspect channels offer spectral flux peak by enhancing the radiative transfer at channel regions *k*Λ/*π* → 1 (Fig. 5(a)), which is weaker for gratings with lower filling ratios; see Fig. 5(b). The latter, on the other hand, results in a stronger radiative flux at a smaller **k** at a higher frequency as shown in Fig. 5(c). The *k*_*x*_-*k*_*y*_ plot also shows that 2D gratings provide the same enhancement in both *x* and *y* directions for thermal emission with uniform polarization in both directions.

## Discussion

**Figure 6:**
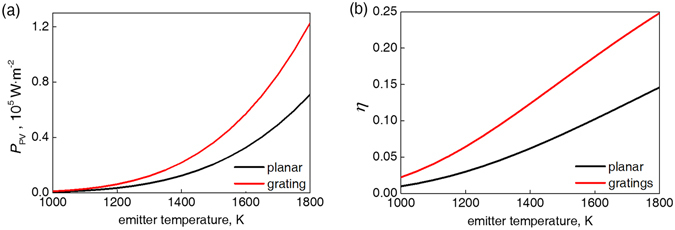
Comparison of (**a**) output power *P*_PV_ and (**b**) conversion efficiency *η* for GaSb with and without grating (Λ = 200 nm, *a* = 150 nm, and *h* = 200 nm).

To evaluate the influence of added surface structures from the standpoint of TPV application, we calculated the manifested electrical power *P*_PV_ and conversion efficiency *η* using Equations (9) and (10), which include both radiative loss (for photons with lower energy than the GaSb bandgap) and thermal loss (for photons with higher energy than the bandgap) but not the electrical loss resulting from EHP recombination. Figure 6 shows a significant enhancement in both output power and conversion efficiency. The grating structure with optimal parameters (Λ = 200 nm, *a* = 150 nm, and *h* = 200 nm) results in an average of 75.4% enhancement in the output power *P*_PV_ for the studied emitter temperatures (1000–1800 K). At 1800 K, the conversion efficiency rises from the original 14.5% to 24.8%. The maximum output power and efficiency is reached by the same set of grating parameters.

The proposed design offers other practical benefits, as surface structures with very high aspect ratio channels can be manufactured by deep etching III–V semiconductors^16, 29^, which might not be possible for metallic and other types of dielectric materials. Further, the microstructures we proposed are located on the cold side of the TPV device instead of on the thermal emitter where fine structures suffer severe thermal stability issues at very high temperatures. Finally, the strategy we discussed can be easily generalized to TPV devices operating at different temperatures by choosing from a wide range of infrared plasmonic emitter materials^11^ and semiconductors with different bandgaps^30^, including ternary and quaternary types because of their very similar refractive index model.

The radiative transfer analysis was simplified by omitting the surface electrical contacts (such as an ultra-thin ITO layer or Au nano-electrodes)^7^ and the conductive substrates necessary for extraction of the generated charges. The surface electrodes should have a negative impact due to the surface wave coupling to the emitter and losses in metallic materials, while the conductive substrate normally improves the efficiency by limiting long-wavelength radiative transfer as a back-reflector^10, 11^. Also, the nano patterning of the PV cells is likely to increase the electrical losses via surface recombination^31^ but this effect is difficult to predict due to the complexity of determining the spatial generation and recombination of EHPs in this scenario, so this effect is outside the scope of this analysis. For practical applications, methods such as surface passivation^32^ may be used to reduce the electrical losses for deeply etched group III–V semiconductors.

In conclusion, we present an “anti-reflection” strategy for group III–V cells used for nano-gap TPV devices by applying simple 2D rectangular gratings on the cell surface. When coupled with an IR plasmonic emitter, adding grating structures on GaSb results in greatly increased spectral radiative flux above the cell’s bandgap with the maximal spectral enhancement at 2.8 times and slightly suppressed radiative flux below the bandgap, which altogether leads to significant improvement of the output power and conversion efficiency of the TPV system. The manipulated spectral radiative flux is due to both the effective medium effect and the resonance in high aspect ratio grating channels, depending on the grating size parameters. The result of this work shows a novel and practical strategy for higher system efficiency and power output for a nano-gap TPV device.

## Methods

### Calculation of near-field radiative transfer

The spectral radiative heat flux *q*(*ω*) from a semi-infinite emitter (designated 1) to a semi-infinite absorber (designated 2) across a vacuum gap (designated 0) at angular frequency *ω* and temperature *T* is calculated by a rigorous formulation based on the fluctuational-dissipation theorem^5^:

1 $$q(\omega )=\frac{{\rm{\Theta }}(\omega ,{T}_{1})-{\rm{\Theta }}(\omega ,{T}_{2})}{{\pi }^{2}}{\int }_{0}^{\infty }\xi ({k}_{//},\omega ){k}_{//}d{k}_{//}$$

where ${k}_{//}=\sqrt{{k}_{x}^{2}+{k}_{y}^{2}}$ is the lateral component of wave vector, ${\rm{\Theta }}(\omega ,T)=\hslash \omega /[\exp (\hslash \omega /{k}_{{\rm{B}}}T)-1]$ is the mean energy of a Planck oscillator at the thermal equilibrium state, *ħ* is the reduced Planck constant, and *k*_B_ is the Boltzmann constant. The expression of the energy transmission coefficient *ξ*(**k**, *ω*) for propagating waves (*k*_//_ < *k*_0_ = *ω*/*c*_0_, where *c*_0_ is the speed of light in vacuum) and evanescent waves (*k*_//_ > *k*_0_), respectively, is determined by^33^

2 $${\xi }_{{\rm{p}}{\rm{r}}{\rm{o}}{\rm{p}}}({k}_{//},\omega )=\sum _{\gamma ={\rm{s}},{\rm{p}}}\frac{(1-{|{R}_{1}^{\gamma }|}^{2})(1-{|{R}_{2}^{\gamma }|}^{2})}{4{|1-{R}_{1}^{\gamma }{R}_{2}^{\gamma }\exp (2i{k}_{z0}d)|}^{2}}$$

3 $${\xi }_{{\rm{evan}}}({k}_{//},\omega )=\sum _{\gamma ={\rm{s}},{\rm{p}}}\frac{{\rm{Im}}({R}_{1}^{\gamma }){\rm{Im}}({R}_{2}^{\gamma })\exp (-2\mathrm{Im}({k}_{z0})d)}{{|1-{R}_{1}^{\gamma }{R}_{2}^{\gamma }\exp (2i{k}_{z0}d)|}^{2}}$$

where *γ* = s, p marks the polarization states and *k*_z0_ is the *z*-component of the wave vector in the vacuum gap. The relative permittivity *ε* of material can be uniaxial anisotropical:

4 $${\varepsilon }_{i}=[\begin{matrix}{\varepsilon }_{i,//} &  & \\  & {\varepsilon }_{i,//} & \\  &  & {\varepsilon }_{i,\perp }\end{matrix}]$$

When the emitter and the absorber are both homogenous bulks, the field reflection coefficients *R*_1_ and *R*_2_ can be simplified into the Fresnel reflection coefficient at the vacuum gap interfaces *r*_01_ and *r*_02_, which for a possibly anisotropic medium has the formulation^9^

5 $${r}_{ij}^{{\rm{s}}}=\frac{{k}_{zi}^{{\rm{s}}}-{k}_{zj}^{{\rm{s}}}}{{k}_{zi}^{{\rm{s}}}+{k}_{zj}^{{\rm{s}}}},\,{r}_{ij}^{{\rm{p}}}=\frac{{\varepsilon }_{j,//}{k}_{zi}^{{\rm{p}}}-{\varepsilon }_{i,//}{k}_{zj}^{{\rm{p}}}}{{\varepsilon }_{j,//}{k}_{zi}^{{\rm{p}}}+{\varepsilon }_{i,//}{k}_{zj}^{{\rm{p}}}}$$

where for given *k*_//_ at *ω*, *k*_*z*_ for s- and p-polarization is determined by

6 $${k}_{z,i}^{{\rm{s}}}=\sqrt{{\varepsilon }_{i,//}{k}_{0}^{2}-{k}_{//}^{2}},{k}_{z,i}^{{\rm{p}}}=\sqrt{{\varepsilon }_{i,//}{k}_{0}^{2}-\frac{{\varepsilon }_{i,//}}{{\varepsilon }_{i,\perp }}{k}_{//}^{2}}$$

For the effective medium calculation in this work, the absorber consists of 2 layers, the surface layer *a* with thickness *t* and the substrate *b*, which is semi-infinite. For this case, the reflection coefficient *R* is written as ref. 34

7 $${R}_{2}^{\gamma }=\frac{{r}_{0a}^{\gamma }+{r}_{ab}^{\gamma }\,\exp (2i{k}_{za}^{\gamma }t)}{1+{r}_{0a}^{\gamma }{r}_{ab}^{\gamma }\,\exp (2i{k}_{za}^{\gamma }t)}$$

Compared to other formulations^9, 33^, in this work, we have the transmission terms *T*_1_ = *T*_2_ = 0 because both the emitter and the absorber are opaque and semi-infinite.

In the case of using EMT as an approximation for the GaSb surface gratings, the permittivity of the effective layer is determined by refs 27, 28 and 35

8 $${\varepsilon }_{{\rm{eff}},\perp }=f{\varepsilon }_{{\rm{GaSb}}}+(1-f){\varepsilon }_{0},\,{\varepsilon }_{{\rm{eff}},//}=\frac{{\varepsilon }_{0}{\varepsilon }_{{\rm{GaSb}}}}{(1-f){\varepsilon }_{{\rm{GaSb}}}+f{\varepsilon }_{0}}$$

Equations (1)–(8) thus complete the radiative transfer calculation in a one-dimensional system. Further, to avoid the dependence of temperature, we define the normalized spectral flux as $\bar{q}(\omega )=q(\omega )/({\rm{\Theta }}(\omega ,{T}_{1})-{\rm{\Theta }}(\omega ,{T}_{2}))$ as shown in Fig. 1(a).

The radiative transfer power and efficiency of the PV cell is calculated approximately^36, 37^ by modelling the PV cell as an ideal thermodynamic diode operating at a voltage approaching the theoretical limit *V*_0_ = ℏ*ω*_g_(1 − *T*_2_/*T*_1_)/*e*. The radiative power exchange, *P*_rad_, and the electricity generation, *P*_PV_, can then be calculated as

9 $${P}_{{\rm{rad}}}={\int }_{0}^{\infty }\frac{\hslash \omega }{\exp (\hslash \omega /{k}_{{\rm{B}}}{T}_{1})-1}\bar{q}(\omega )d\omega -{\int }_{{\omega }_{{\rm{g}}}}^{\infty }\frac{\hslash \omega }{\exp [\hslash (\omega -{\omega }_{0})/{k}_{{\rm{B}}}{T}_{2}]-1}\bar{q}(\omega )d\omega $$

10 $${P}_{{\rm{PV}}}={\int }_{{\omega }_{{\rm{g}}}}^{\infty }\hslash {\omega }_{0}({(\exp (\hslash \omega /{k}_{{\rm{B}}}{T}_{1})-1)}^{-1}-{(\exp (\hslash (\omega -{\omega }_{0})/{k}_{{\rm{B}}}{T}_{2})-1)}^{-1})\bar{q}(\omega )d\omega $$

where *ω*_0_ = *eV*_0_/*ℏ*. The resultant energy conversion efficiency, *η* = *P*_PV_/*P*_rad_, accounts for the radiative and the thermal losses but not for the electrical loss in a non-ideal cell. In our numerical calculation, $\bar{q}(\omega )$ is integrated within 0.1–3.0 × 10^15^ rad/s. Material refractive indices, unless otherwise stated, are taken from the handbook by Palik^38^.

### RCWA model

Near-field radiative heat flux involving periodic structures is determined by combining the RCWA method and the fluctuational-dissipation theorem^18, 21, 22, 39–43^. Because of the symmetry of 2D rectangular gratings, the normalized radiative flux is calculated by integrating *ξ* over the lateral wave vector in a quarter of the first Brillouin zone 0 ≤ *k*_*x*_, *k*_*y*_ < *π*/Λ:

11 $$\bar{q}(\omega )=\frac{1}{2{\pi }^{3}}{\int }_{0}^{\pi /{\rm{\Lambda }}}{\int }_{0}^{\pi /{\rm{\Lambda }}}\xi ({k}_{x},{k}_{y},\omega )d{k}_{x}d{k}_{y}$$

The exchange function *ξ*(*k*_*x*_, *k*_*y*_, *ω*) is determined by

12 $$\xi ({k}_{x},{k}_{y},\omega )={\rm{tr}}({\bf{D}}{{\bf{W}}}_{1}{{\bf{D}}}^{\ast }{{\bf{W}}}_{2})$$

13 $${\bf{D}}={({\bf{I}}-{{\bf{S}}}_{1}{{\bf{S}}}_{2})}^{-1}$$

14 $${{\bf{W}}}_{1}={{\boldsymbol{\Sigma }}}_{-1}^{{\rm{pw}}}-{{\bf{S}}}_{1}{{\boldsymbol{\Sigma }}}_{-1}^{{\rm{pw}}}{{{\bf{S}}}_{1}}^{\ast }+{{\bf{S}}}_{1}{{\boldsymbol{\Sigma }}}_{-1}^{{\rm{ew}}}-{{\boldsymbol{\Sigma }}}_{-1}^{{\rm{ew}}}{{{\bf{S}}}_{1}}^{\ast }$$

15 $${{\bf{W}}}_{2}={{\boldsymbol{\Sigma }}}_{1}^{{\rm{pw}}}-{{{\bf{S}}}_{2}}^{\ast }{{\boldsymbol{\Sigma }}}_{1}^{{\rm{pw}}}{{\bf{S}}}_{2}+{{{\bf{S}}}_{2}}^{\ast }{{\boldsymbol{\Sigma }}}_{1}^{{\rm{ew}}}-{{\boldsymbol{\Sigma }}}_{1}^{{\rm{ew}}}{{\bf{S}}}_{2}$$

16 $${{\bf{S}}}_{1}={{\bf{R}}}_{1}({k}_{x},{k}_{y},\omega ),\,{{\bf{S}}}_{2}={e}^{i{{\bf{k}}}_{z0}d}{{\bf{R}}}_{2}({k}_{x},{k}_{y},\omega ){e}^{i{{\bf{k}}}_{z0}d}$$

**R**_1_ and **R**_2_ are the reflection matrices calculated by RCWA, with dimensions of 2(2*N*_ord,*x*_ + 1) (2*N*_ord,*y*_ + 1), where *N*_ord_ is the maximum diffraction order for each direction. **I** is the identity matrix. The asterisk is the Hermitian transpose of the matrix. ${{\boldsymbol{\Sigma }}}_{\pm 1}^{\mathrm{pw}/\mathrm{ew}}$ is the operator for propagating/evanescent waves defined by

17 $${{\boldsymbol{\Sigma }}}_{\pm 1}^{\mathrm{pw}/\mathrm{ew}}={{\bf{k}}}_{z0}^{\pm 1}{{\boldsymbol{\Pi }}}^{\mathrm{pw}/\mathrm{ew}}$$

18 $${{\boldsymbol{\Pi }}}^{{\rm{p}}{\rm{w}}}=\frac{1}{2}(1+{\rm{s}}{\rm{g}}{\rm{n}}({k}_{0}^{2}-{k}_{//}^{2})),{{\boldsymbol{\Pi }}}^{{\rm{e}}{\rm{w}}}=\frac{1}{2}(1-{\rm{s}}{\rm{g}}{\rm{n}}({k}_{0}^{2}-{k}_{//}^{2}))$$

Eqs (11)–(18) completes the RCWA calculations for near-field radiative heat flux. Implementation of RCWA follows the framework by Li^44, 45^, which improves the convergence of the original RCWA formulation^46^. For all RCWA calculations in this work, we set *N*_ord,*x*_ = *N*_ord,*y*_ = 12, resulting in matrices with dimensions of 1250 × 1250. The numerical integration in Equation (10) uses 24 × 24 segments. Doubling the diffraction orders or the segments for wave numbers has been found to cause less than 2% relative change in spectral flux results. The computation is done on a Dell Workstation with Intel Xeon E5-2630 v3 CPUs (2.40 GHz) with a calculation for each *ω* assigned to a single thread with 2GB RAM. Computation for a single case takes 36–40 hours to finish.

**Figure 7:**
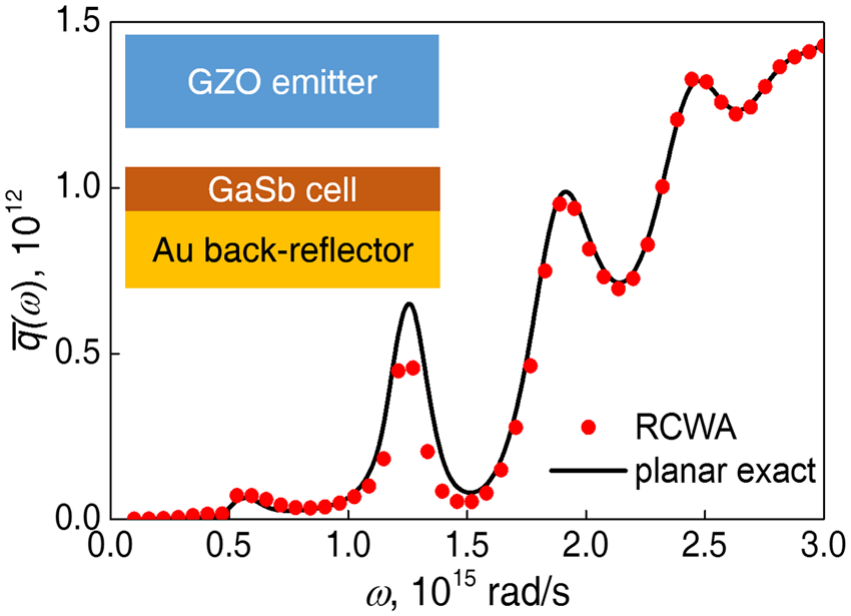
Comparison of the RCWA result and the accurate solution for near-field radiative transfer. The thickness of GaSb is 200 nm. Vacuum gap *d* = 200 nm.

**Figure 8:**
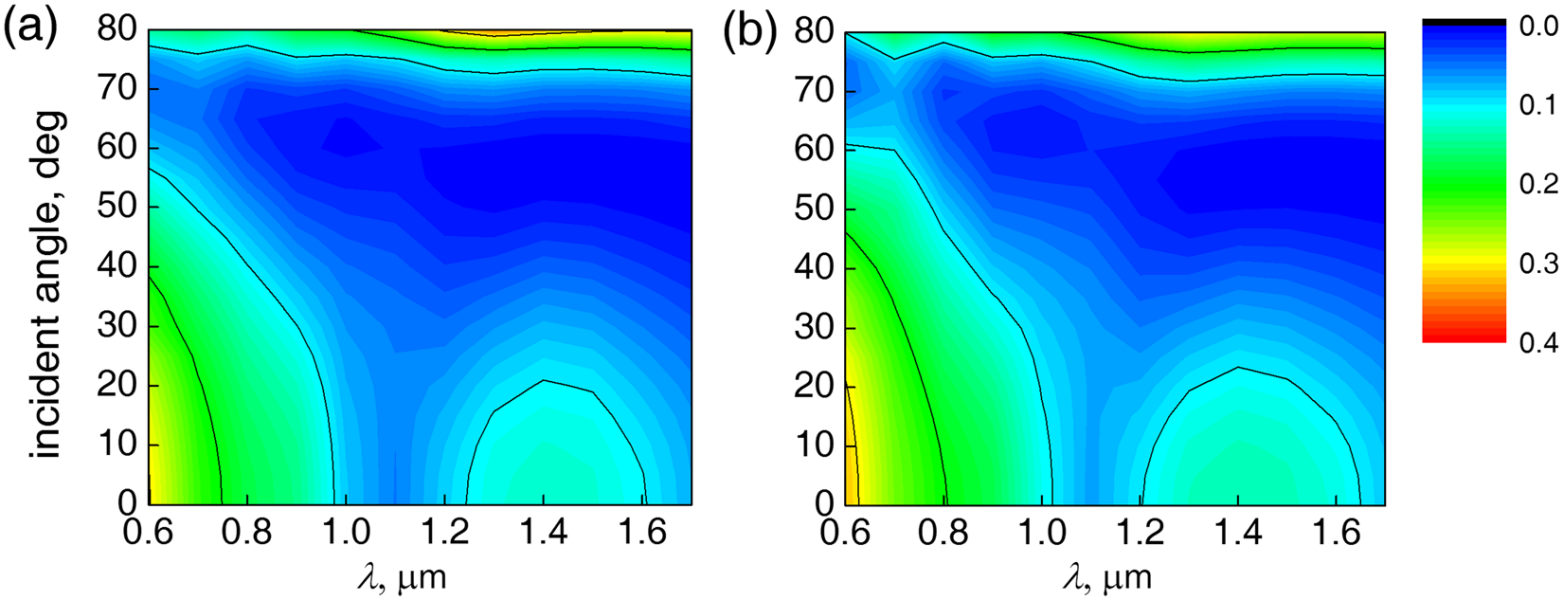
Far-field p-pol reflectance of GaSb with grating parameters Λ = 400 nm, *a* = 320 nm, and *h* = 200 nm. (**a**) Lumerical FDTD. (**b**) RCWA.

For examination of the numerical program, we compared the RCWA results with the exact solution of normalized radiative heat flux for a case with an ultrathin GaSb layer on a gold back reflector in the hope to enhance the resonance and cause energy exchange with a larger lateral wave vector to examine the program’s accuracy (Fig. 7). The agreement of the results is satisfactory. To examine the accuracy when handling GaSb grating structures, the far-field reflection is computed from the reflection matrix **R**_2_ and compared to the result of a commercial software (Lumerical FDTD solutions), which applies the finite-difference time domain method. The error between FDTD and RCWA is within 5% for incident angle *θ* ≤ 80° and 0.6 < *λ* < 1.7 μm (Fig. 8).

## Electronic supplementary material

Supplimentary information
