# Supplementary material for: Simple Rectangular Gratings as a Near-Field “Anti-Reflection” Pattern for GaSb TPV Cells
Source: Sci Rep. 2017 Apr 21;7:1026. doi: 10.1038/s41598-017-01197-2 (PMC5430919; doi:10.1038/s41598-017-01197-2)
Supplement: Supplementary file 1 — Supplimentary information [file 41598_2017_1197_MOESM1_ESM.pdf]

Supplementary information

Simple rectangular gratings as a near-field "anti-reflection" pattern for GaSb TPV cells

Haitong Yu,[1] Dong Liu,[2] Zhen Yang,[1] Yuanyuan Duan[1 *]

[1]Key Laboratory of Thermal Science and Power Engineering of Ministry of Education, Beijing Key Laboratory for CO_2 Utilization and Reduction Technology, Tsinghua University, Beijing 100084, China

[2]MIIT Key Laboratory of Thermal Control of Electronic Equipment，School of Energy and Power Engineering, Nanjing University of Science and Technology, Nanjing 210094, China

*Contact author: yyduan@tsinghua.edu.cn

Material refractive indices. The refractive index of 6wt% Ga:ZnO (GZO)[1] is shown in Fig. S1. The permittivity is determined by the Drude-Lorentz model:

$$\varepsilon(\omega)=\varepsilon_{\infty}-\frac{\omega_{\mathrm{p}}^{2}}{\omega\left(\omega+i\Gamma_{\mathrm{p}}\right)}+\frac{f_{1}\omega_{1}^{2}}{\omega_{1}^{2}-\omega^{2}-i\omega\Gamma_{1}} \quad \text{(S1)}$$

and parameters for GZO are ε_{∞}=2.475, ω_{p}=1.927 eV, Γ_{p}=0.117 eV, f_{l}=0.866 eV, ω_{l}=4.850 eV, Γ_{l}=0.029 eV.

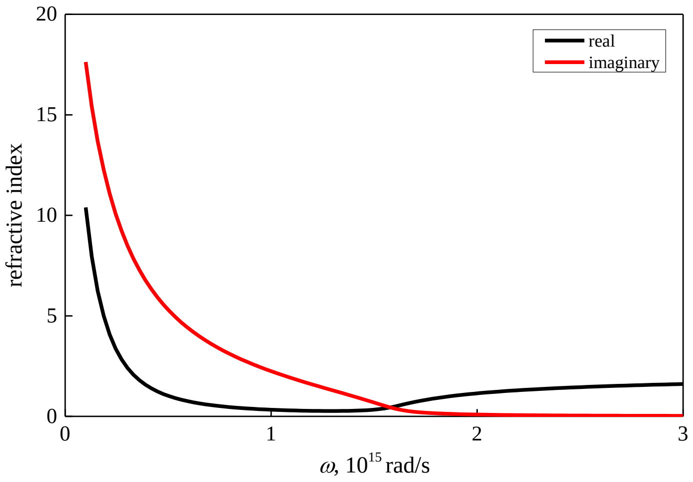

Figure S1. Refractive index of GZO

Real part of the refractive index of GaSb[2] are plotted in Fig. S2 in comparison with the uniaxial parameters calculated by EMT. In the studied spectral range, GaSb has a refractivity of around 4-5 which results in high Fresnel reflection coefficients. The effective indices for GaSb gratings as predicted by EMT, on the other hand, are much smaller and help to suppress the reflection.

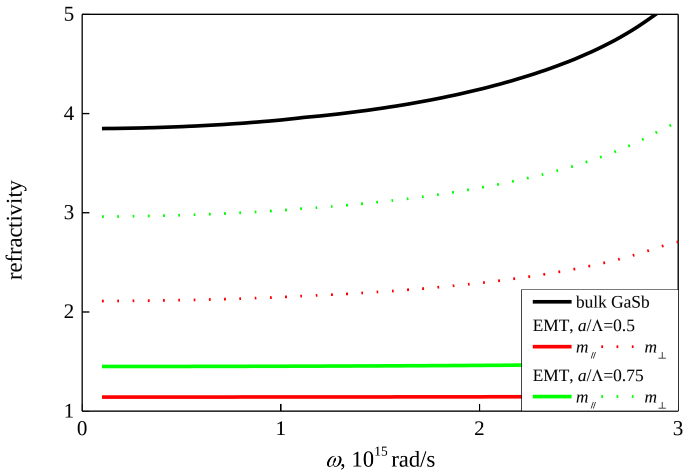

Figure S2. Real part of refractive index of GaSb and uniaxial effective indices.

Discrepancy between RCWA and EMT for larger periods. We show the comparison between RCWA and EMT results for Λ=400 nm (Fig. S3). Compared with the case for Λ=200 nm (Fig. 3b), increasing the period results in larger discrepancy of EMT from rigorous solution, and again is most obvious for larger *a*/Λ.

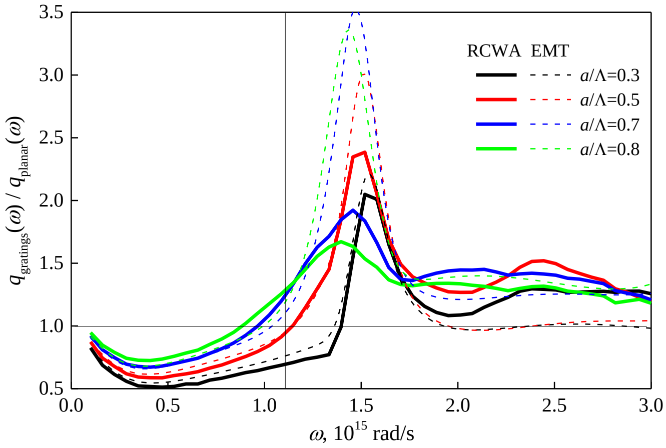

Figure S3. Comparison between RCWA and EMT results for Λ=400 nm.

Influence of grating heights. We study the influence of h under parameters Λ=200 nm, a=150 nm and d=200 nm. EMT prediction (Fig. S4a) shows the maximum enhancement is reached at h=200 nm and that increasing h will shift the peak to lower frequency. The RCWA calculation for h=100, 200 and 300 nm proved the above predictions (Fig. S4b), although EMT overestimates the peak enhancement compared with the rigorous solution.

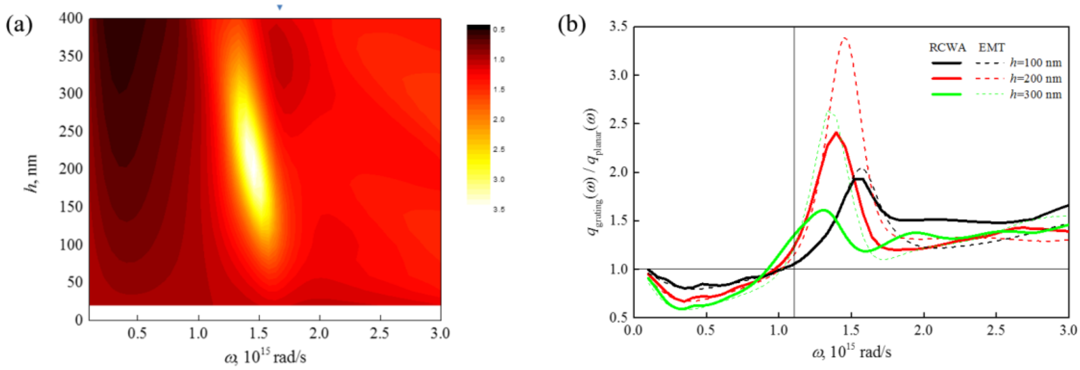

Figure S4. Influence of grating heights h.

References

1. Kim, J., Naik, G. V., Emani, N. K., Guler, U. & Boltasseva, A., Plasmonic Resonances in Nanostructured Transparent Conducting Oxide Films. *IEEE J. Sel. Top. Quant.* **19** 4601907 (2013).
2. Gonzalez-Cuevas, J. A., Refaat, T. F., Abedin, M. N. & Elsayed-Ali, H. E., Calculations of the temperature and alloy composition effects on the optical properties of $Al_xGa_{1-x}As_ySb_{1-y}$ and $Ga_xIn_{1-x}As_ySb_{1-y}$ in the spectral range 0.5-6 eV. *J. Appl. Phys.* **102** 14504 (2007).
